# High-resolution versus standard-resolution cardiovascular magnetic resonance perfusion imaging for the detection of coronary artery disease

**DOI:** 10.1186/1532-429X-14-S1-O89

**Published:** 2012-02-01

**Authors:** Manish Motwani, Neil Maredia, Timothy Fairbairn, Sebastian Kozerke, Aleksandra Radjenovic, John P Greenwood, Sven Plein

**Affiliations:** 1 Multidisciplinary Cardiovascular Research Centre & Leeds Institute of Genetics, Health and Therapeutics, University of Leeds, Leeds, UK; 2 Institute for Biomedical Engineering, University and ETH Zurich, Zurich, Switzerland; 3 NIHR Leeds Musculoskeletal Biomedical Research Unit, University of Leeds, Leeds, UK

## Summary

This study compared the diagnostic accuracy of high-resolution and standard-resolution cardiovascular magnetic resonance (CMR) perfusion imaging in patients with suspected coronary artery disease (CAD).

## Background

Although accelerated high-spatial-resolution CMR perfusion imaging has recently been shown to be clinically feasible, there has not yet been a direct comparison with standard-resolution methods. We hypothesised that higher spatial resolution detects more subendocardial ischemia and leads to greater diagnostic accuracy for the detection of angiographically defined CAD.

## Methods

A total of 111 patients with suspected CAD were prospectively recruited. All patients underwent two separate perfusion CMR studies on a 1.5 Tesla CMR scanner (Intera CV, Philips Healthcare, Best, the Netherlands), one with standard-resolution (2.5 x 2.5mm in-plane resolution) and one with high-resolution (1.6 x 1.6mm in-plane resolution) acquisition. High-resolution acquisition was facilitated by eight-fold k-t broad linear speed up technique (BLAST) acceleration. Two observers visually graded perfusion in each myocardial segment on a 4-point scale. Segmental scores were summed to produce a perfusion score for each patient. All patients underwent invasive coronary angiography. Significant CAD was defined as a coronary artery stenosis of ≥ 50% diameter on quantitative coronary angiography.

## Results

**Figure 1:**
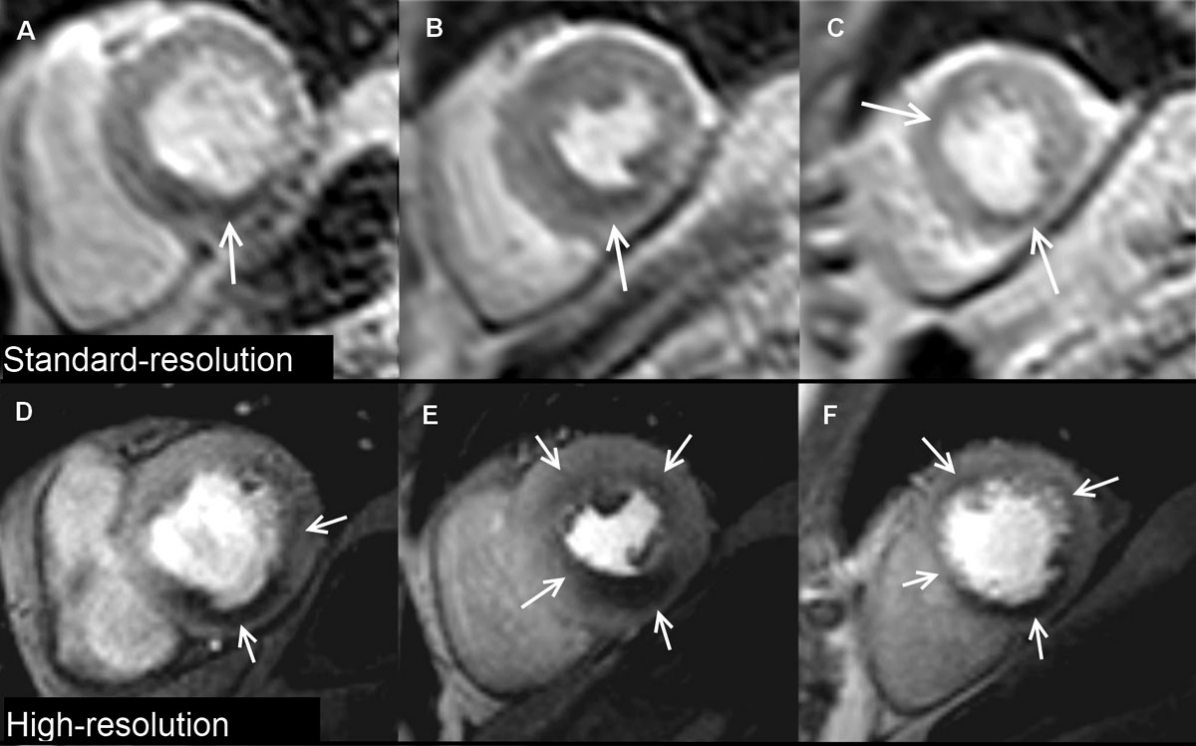
Case Example Standard and high-resolution stress perfusion CMR images in a patient with three-vessel coronary artery disease. Standard-resolution shows perfusion defects (white arrows) in the basal inferior (A), mid inferior, mid inferoseptal (B), apical anterior and apical inferior segments (C). High-resolution shows a similar distribution of perfusion defects but demonstrates additional ischemia in the basal lateral (D), mid anterior and mid anterolateral segments (E) with a circumferential defect in the apical slice (F). Perfusion defects are also better delineated at high-resolution and the transmural extent of ischemia more clearly seen.

**Figure 2:**
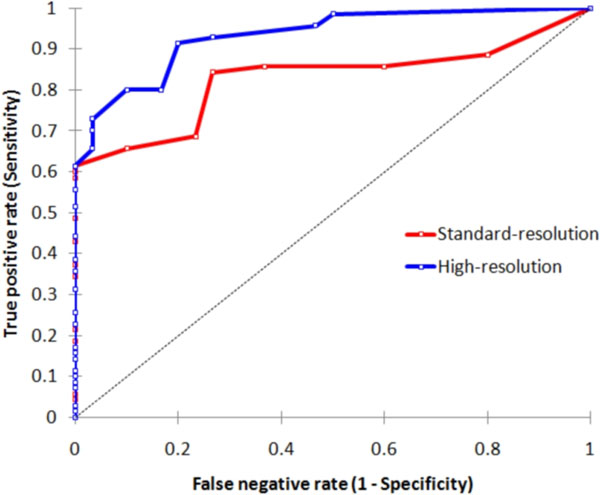
Receiver-Operator Characteristic Curves. Standard and high-resolution perfusion CMR both had a high diagnostic accuracy for the detection of coronary artery disease but the high-resolution technique was superior. The areas under the curve were 0.83 (95% CI: 0.75-0.91) for standard-resolution and 0.93 (95% CI: 0.88-0.98) for high-resolution (p<0.001).

CMR data were successfully obtained in 100 patients. A typical example is shown in Figure 1. In patients with CAD (n=70), more segments were determined to have subendocardial ischemia with high-resolution acquisition than with standard-resolution acquisition (279 vs.108; p<0.001). High-resolution acquisition had a greater diagnostic accuracy than standard-resolution acquisition for identifying single-vessel disease (area under the curve [AUC]: 0.88 vs. 0.73; p<0.001) or multi-vessel disease (AUC: 0.98 vs. 0.91; p=0.002) and overall (AUC: 0.93 vs. 0.83; p<0.001) (Figure 2).

## Conclusions

Our study shows that high-resolution CMR perfusion imaging has greater diagnostic accuracy than standard-resolution acquisition for the detection of CAD in both single and multi-vessel disease and detects more subendocardial ischemia.

## Funding

S.P is funded by a British Heart Foundation fellowship (FS/10/62/28409).

S.P and J.P.G received an unrestricted educational research grant from Philips Healthcare.

